# Application of an Acrylic Polymer and Epoxy Emulsion to Red Clay and Sand

**DOI:** 10.3390/polym13193410

**Published:** 2021-10-04

**Authors:** Sung-Sik Park, Jung-Shin Lee, Keun-Byoung Yoon, Seung-Wook Woo, Dong-Eun Lee

**Affiliations:** 1 Department of Civil Engineering, Kyungpook National University, 80 Daehakro, Bukgu, Daegu 41566, Korea; sungpark@knu.ac.kr (S.-S.P.); geowsw@knu.ac.kr (S.-W.W.); 2 Intelligent Construction Automation Center, Kyungpook National University, Global Plaza 905, 80 Daehakro, Bukgu, Daegu 41566, Korea; jhjs14@knu.ac.kr; 3 Department of Polymer Science and Engineering, Kyungpook National University, Daegu 41566, Korea; kbyoon@knu.ac.kr; 4 School of Architecture, Civil, Environment and Energy Engineering, Kyungpook National University, 80 Daehakro, Bukgu, Daegu 41566, Korea

**Keywords:** red clay, sand, acrylic polymer, epoxy, unconfined compressive strength

## Abstract

The use of nontraditional soil stabilizers increases. Various new soil binding agents are under study to augment renewability and sustainability of an earth structure. However, despite increasing interest involved in red clay, there is minimal research investigating the stabilizing red clay with polymer. This paper presents the findings obtained by applying the acrylic polymer and epoxy emulsion as binding agent for red clay and that for sand. The epoxy–hardener ratio, amount of epoxy emulsion, and amount of polymer aqueous solution were manipulated to quantify their effects on red clay and sand, respectively. After compacting a pair of cylindrical samples of which diameter and height are 5 cm and 10 cm, respectively, it is cured for 3 and 7 days in a controlled condition. Each pair is produced to represent the engineering performance at each data point in the solution space. An optimal composition of the binding agents for red clay and that for sand mixture are identified by experimenting every data point. In addition, given lime into each sample, the maximum unconfined compressive strength (UCS) endured by red clay sample and that by sand sample are 2243 and 1493 kPa, respectively. The UCS obtained by the sample mixed with clay and sand reaches 2671 kPa after seven days of curing. It confirms that the addition of lime remarkably improves the UCS. When the clay–sand mixture, of which the ratio is 70:30, includes 5% lime, the UCS of the mixture outperforms. Indeed, these findings, i.e., the optimal proportion of components, may contribute to the increase of initial and long-term strength of an earth structure, hence improving the renewability and sustainability of the earth construction method.

## 1. Introduction

Soil stabilization is an important technology in the civil construction field. Compaction, reinforcement, nailing, chemical mixing (i.e., lime, cement, polymer, etc.), and grouting are well accepted soil stabilization methods that improve engineering characteristics of soil [1]. They may be classified into either chemical, mechanical, and/or physical stabilization [2]. Among them, lime and cement mixing method classified as a chemical method is most popular for stabilization. Lime has been used for several decades, and historical records indicate that it was applied to the construction of pyramids [3]. Cement demands low cost but manifests high strength and resistance against water and temperature. However, although cement is a great soil stabilizer, recent studies pursue developing a nontraditional stabilizer by using salts, acids, enzymes, lignosulfonates, petroleum emulsions, polymers, and resins [4,5,6,7,8]. It is attributed to the fact that a large CO_2_ is emitted during the cement producing process [9,10]. In addition, the Ca(OH)_2_ of the cement increases the soil pH [11]. The quality of water and land vegetation could be affected by the alkaline water. A nontraditional stabilizer is under study to complement these issues by replacing the cement. Especially, the uses of nontraditional stabilizers, polymers, increases in these days [12]. It is known that polymers, which are a combination of long molecular chains, improve the strength of soil attributed to the formation of a bond between clay particles and polar end groups of polymers [13].

Ryu et al. tried to apply acrylic polymer [14]. They observed that their binding agent enhanced the strength of red clay more than water and cement. Furthermore, Kim et al. studied about the early strength of the red clay bound with acrylic polymer for the autonomous rammed earth construction [15]. They applied an epoxy emulsion to improve early strength. The red clay samples to which the emulsion and the polymer were added showed higher early strength than those with added Portland cement and polymer. The early strength of red clay, which was mixed with the epoxy emulsion and polymer, reached 3.2 MPa in 24 h. The combination of the epoxy emulsion and polymer improved the strength of red clay. In general, the epoxy resin has been applied to numerous construction fields such as soil stabilization and cement concrete [16,17,18]. Additionally, lots of research has been performed on the impact of epoxy resin on the construction material behavior [19,20]. Moreover, in some studies, epoxy resin has applied for binding a sand [21,22]. Anagnostopoulos et al. [23] studied the optimal ratio of epoxy and hardener in terms of strength. Rahmannejad et al. [24] examined effects of water content and curing time on the strength of epoxy resin samples.

A few recent studies with clay and polymer report the enhancement of mechanical performances of soil structure [25,26,27] and investigate the copolymerization with red clay [28]. However, this study evaluates the polymer as a soil stabilization binder for ground reinforcement. The more the interest in the eco-friendly material increases, the more the demand for red clay in civil engineering sector increases. Indeed, the red clay is well accepted for paving trail paths due to aesthetic purposes. Thus, this study made three types of samples with the polymer and epoxy emulsion: a sand sample, red clay sample, and sand and red clay mixture sample. Unlike existing studies applying binders to red clay bricks, this study uses both sand and red clay to evaluate the samples because most of the civil engineering job sites are exposed to either red clay or sand. The acrylic polymer is used for soil stabilizer. From the sand and red clay samples, effects of epoxy–hardener ratio, epoxy emulsion content, and polymer solution were evaluated to find the optimal composition for a sandy clay sample. Additionally, lime was added to the sandy clay sample to enhance the early strength. Lime is a hygroscopic material and is applied for soil stabilization; however, excess lime causes cracking and expansion of the sample, so only a small amount was added. The evaluation of the samples was conducted through the compression test, and the microstructure analysis was analyzed through a scanning electric microscope (SEM).

## 2. Materials and Methods

### 2.1. Materials

#### 2.1.1. Sand and Red Clay

**Figure 1:**
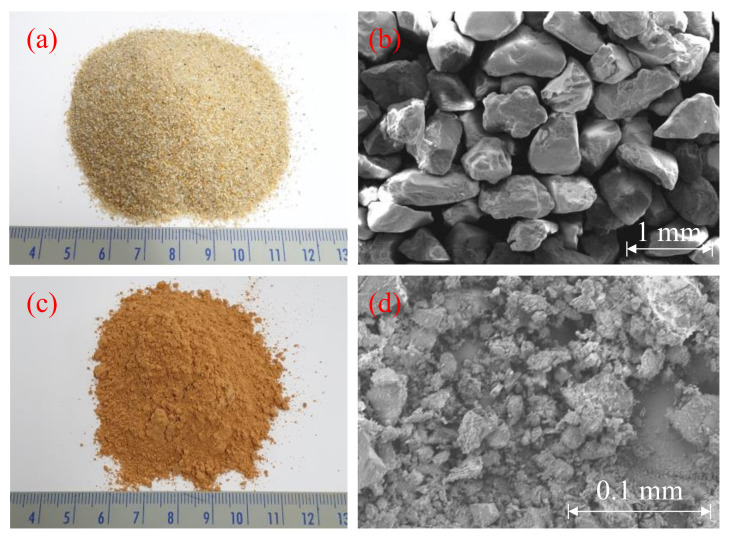
Optical photo from the camera and the image of the SEM ((**a**,**b**): Jumunjin sand, (**c**,**d**): Red clay).

This study used Jumunjin sand for sample preparation. Jumunjin sand is used as the standard sand in Korea, and many studies were conducted with this sand. Its specific gravity is 2.65, and the sand is classified as poorly graded sand (SP), according to the Unified Soil Classification System (USCS). The optical photo from a camera and that from an image obtained by the SEM are shown in (a) and (b) of Figure 1, respectively. The chemical components of the sand are listed in Table 1; X-ray fluorescence was used for component analysis. It contains >87% of silica (SiO_2_). Because of a high portion of silica, the SEM image mainly shows angular particles.

**Figure 2:**
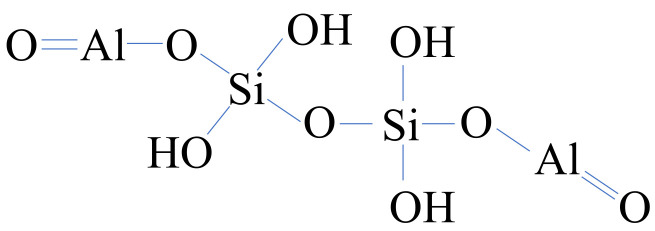
Structural formula of the halloysite group clay.

Red clay was obtained from Hwangtomyungga (Seoul, Korea). The particle size of the red clay was so varied that it had to be sieved through a sieve with a 0.20 mm passing mesh. The optical photo from a camera and an image from the SEM are shown in Figure 1c,d. Its specific gravity is 2.71, and plasticity index is 18. The red clay is classified as CL, according to the USCS. Table 2 showed the chemical components of the red clay. Generally, red clay is classified as a halloysite clay, for which the structural formula is shown in Figure 2. Raw data associated with Table 1 and Table 2 and Figure 1 were collected by the authors. However, the data are not presented due to lack of space.

#### 2.1.2. Epoxy and Acrylic Polymer

**Figure 3:**
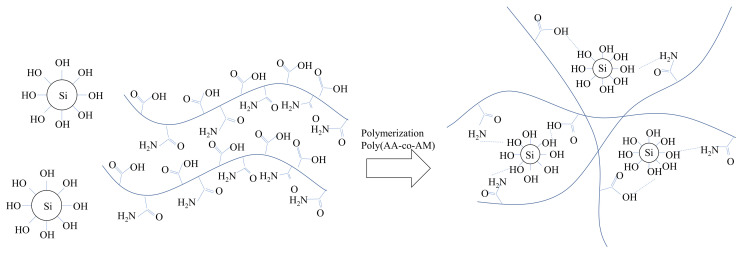
Binding mechanism of the polymer aqueous solution [15].

Epoxy was obtained from Kukdo Chemical (Seoul, Korea), the model was KEM-101-10, and it is waterborne epoxy emulsion. In general, a hardener was required for hardening the epoxy; a lot of epoxy products are sold with their hardener products. This study also used a hardener, KH-700 product, which was obtained from the same company as that of epoxy. The properties of epoxy and hardener shown in Table 3 and Table 4 were provided from the manufacturer, i.e., Kukdo Chemical. As all epoxy emulsions used in this study included a hardener, the epoxy emulsion used afterwards indicates a state of epoxy and hardener mixture. In this study, four epoxy–hardener ratios of 11:1.5, 11:3, 11:6, and 11:9 were evaluated. The acrylic polymer was made with an acrylic acid (AA, >99%), 4,4′-Azobis (4-cyanovaleric acid, >98%), acrylamide (AM, >98%), and ethyl alcohol (>94%). The average molecular weight of the copolymer was 54,000 g/mol. The specific characteristics and manufacturing process of polymer solution are available in Yoon et al. [29]. In this study, 5% polymer aqueous solution, for which the viscosity is 19.02 mPa.s, is used. Figure 3 shows the reaction mechanism of the polymer solution from the previous study [14]. Du et al. elaborates the reaction mechanism with their polymer and clay and deals with the mechanism in detail [30].

### 2.2. Method

#### 2.2.1. Test Method

This study mainly performed the unconfined compression test, according to ASTM D 2166 [31] and KS F 2314 [32]. The machine for compression test is made by Yeon engineering in Korea, and the test compressive rate is 1 mm/min (1%/min). The sample is pressed by moving a bottom plate upward. While doing so, the compression distance and stress are recorded. The UCS of a sample is the average of three UCSs obtained from same type samples with an error less than 5%. From the test, various mechanical properties could be defined, and this study mainly evaluated the UCS and axial strain at the UCS (ε_ucs_). The UCS is the stress at the peak point of a strain–stress curve, and ε_ucs_ is the strain value at the UCS. Additionally, SEM analysis is conducted for the represented samples, and the microstructure of the particles was compared. SU8220 model of Hitachi made in Japan is used. SEM analysis is conducted by shooting electron beam to the surface of the material. The electron beam is combined and conformed the shape image with raster pattern. The samples used for SEM analysis are coated with a platinum and 5.0 kV of acceleration voltage is applied for scanning, and the working distance is arranged in 9–10 mm.

#### 2.2.2. Sample Preparation

**Figure 4:**
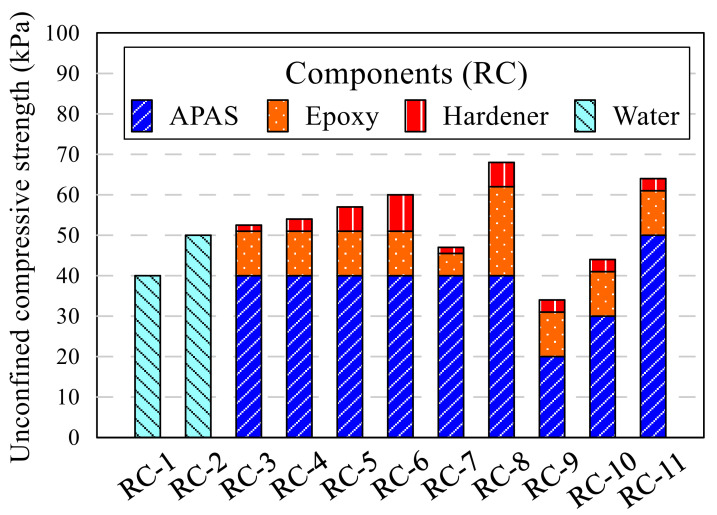
Components of the red clay sample.

**Figure 5:**
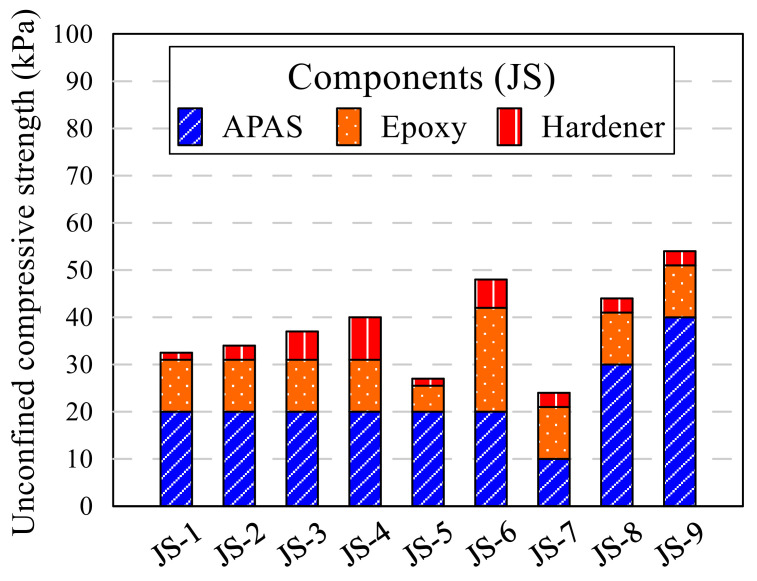
Components of the sand sample.

A cylindrical mold with a diameter of 5 cm and a height of 10 cm was used for sample preparation. All samples were made by dividing into five layers, and each layer was compacted with 1.5 kgf of rammer. Furthermore, compaction was applied to compact the layers. Under compaction was devised by Ladd [33]; this method set a different number of compacting at every layer to make an equal height of each layer. The unit weight of sample was designed with a 17 kN/m^3^. The compacted sample was moved to the curing chamber, which maintains the temperature and humidity as 25 °C and 70%, respectively. The samples in the curing chamber were demolded after 24 h and cured for 2 and 6 days more. The unconfined compression test was conducted after a total of 3 and 7 days curing. The types of samples were divided into two; the red clay sample (RC) and the Jumunjin sand sample (JS). Controlled experiments were performed to identify the best fittest combination by rigorously manipulating the amount of acrylic polymer aqueous solution, the ratio of epoxy, and the ratio of hardener as shown in Table 5 and Figure 4 that defines the component of each RC sample. The amount of acrylic polymer aqueous solution, and ratio of epoxy and hardener were decided at 40 g and 11:3, respectively, as a standard based on a previous experimental study, and the standard amount of epoxy emulsion was 14 g (epoxy 11 g and hardener 3 g), which is 4% of the RC (350 g). By contrast, moist clay gains strength when the moisture in the clay becomes dried, so water was also compared as a binding agent. The components of JS samples are shown in Table 6 and Figure 5. The samples used the same amount of epoxy emulsion and ratio of epoxy and hardener, except amount of the acrylic polymer aqueous solution. The standard amount of the polymer aqueous solution was set as 20 g because of the difference between the sand and red clay in their optimal moisture content. From the results of RC and JS samples, the reasonable binding agent composition was selected as 40 g of acrylic polymer aqueous solution, 11 g of epoxy, and 3 g of hardener for the red clay and sand mixture soil samples. The components of the mixture samples are shown in Table 7. The mixture samples were named as RS, and the ratio of sand in the mixture was added to the end of ID, such as R30 and RS50. In addition, lime was added to the RC and RS samples. 10.5, 17.5, 35, and 52.5 g of lime was applied respectively to the samples; those of weight are 3, 5, 10, and 15% of soil weight, respectively. The letter L was added at the ID of which lime added.

## 3. Results and Discussion

Table 8 presents the results of the UCS test on all RC and JS samples. When unconfined compression test was conducted to all samples, some samples were collapsed without resistance. N.A. indicates those collapsed ones after 3 and 7 days curing period. The strength of RC samples did not develop when the total amount of binder included was low; that of JS sample did not develop when a large amount of binder was included. The effects of each parameter on the UCS are discussed in detail in the succeeding paragraphs.

### 3.1. Effect of the Epoxy–Hardener Ratio

**Figure 6:**
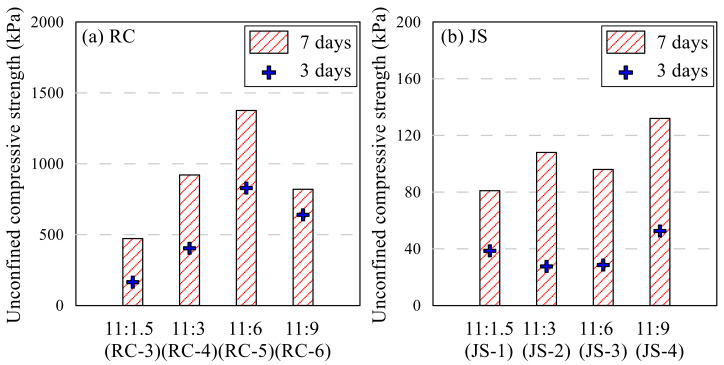
Effect of epoxy–hardener ratio on UCS: RC sample (**a**) and JS sample (**b**).

**Figure 7:**
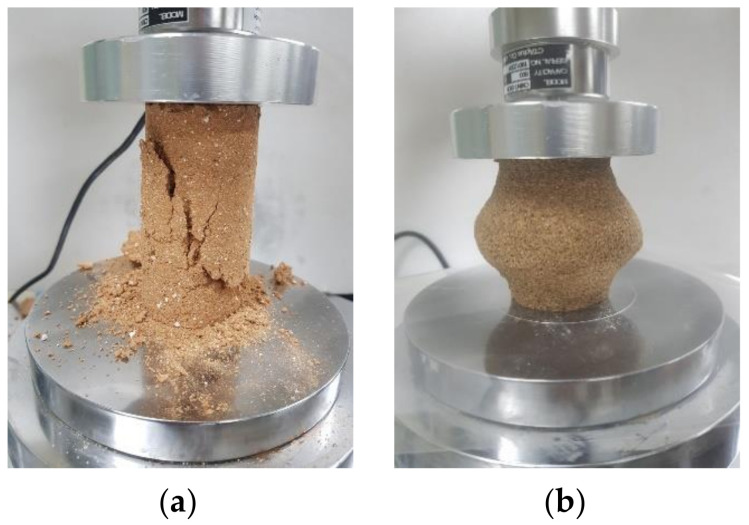
Photo of compressed samples: RC-5 sample (**a**) and JS-4 sample (**b**).

RC-3,4,5,6 and JS-1,2,3,4 samples were made with the same amount of polymer aqueous solution and different epoxy–hardener ratio. The UCS of each type is shown in Figure 6. When the same amount of polymer was used, RC samples showed the highest UCS value at 11:6 for both 3 and 7 days curing period. The strength increased as the portion of the hardener in epoxy emulsion increased from 1.5 to 6; however, when the portion reached 9, the strength decreased to less than 3. Generally, the mechanical properties of epoxy emulsion depend on the ratio of hardener to epoxy. Aziz [34] evaluated the mechanical properties of epoxy resin, according to the hardener content. Two types of common epoxy products were compared on their compressive and bending strength; the highest properties were shown at different hardener contents. D’ALMEIDA [23,35] adjusted the ratio of hardener to epoxy and tested the modulus and strength of the resin and studied the decrease in strength as the ratio of hardener increased. The results of this study show the similar tendency found in Aziz and D’ALMEIDA. The ratio of 11:9 in this study is the result of reflecting these characteristics, and the ratio of 11:6 showed the highest strength for RC. However, in this study, 11:3 was selected as the standard ratio due to efficiency of adding the hardener. The UCS of the sample increased 2 times as the ratio increased from 11:1.5 to 11:3. Strength growth from 3rd to 7th day decreased as the hardener ratio increased, but the final UCS increased. It is thought that this is because the hardener’s hardening action actively proceeds during the first 3 days. The general UCS of JS sample compared with RC sample was poor for all combinations of epoxy and hardener. Entire ε_ucs_ was over 2.0. It was observed that the JS samples could not harden with seven days’ curing and the amounts of polymer aqueous solution and epoxy emulsion were high, regardless of the ratio. Figure 7 shows the photo of RC-5 and JS-4 samples. The RC sample was sheared through a crack, while the JS sample was deformed as its middle part swelled. Clay and sand have a different grain structure. Unlike clay that can absorb moisture, sand cannot absorb moisture well. The amount of water which can be absorbed by the same weight of clay and sand varies considerably. In the case of RC, strength was expressed by absorbing moisture from the epoxy emulsion and polymer aqueous solution, but JS swelled due to a lot of moisture inside.

### 3.2. Effect of the Amount of Epoxy Emulsion

**Figure 8:**
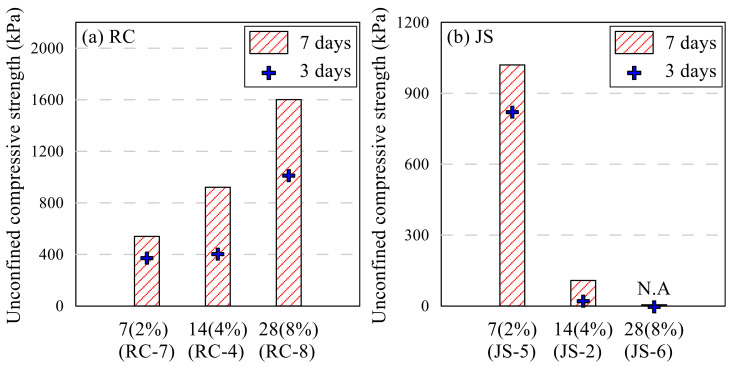
Effects of epoxy emulsion content on UCS: RC sample (**a**) and JS sample (**b**).

About 14 g of epoxy emulsion, which included the epoxy and hardener as 11 g and 3 g, respectively, is 4% of the RC and JS of the samples. Additionally, the RC-7/8 and JS-5/6 samples contained different amounts of epoxy emulsion. RC-7 and JS-5 are made with 2% of the emulsion, and RC-8 and JS-6 are 8%. UCS of RC samples increased as the amount of emulsion increased, however, that of JS samples decreased; the results are shown in Figure 8. These UCS were caused the interior moisture. JS-2 and JS-6, except for JS-5, exceeded the amount of water that Jumunjin sand could contain due to the moisture of the emulsion as the epoxy emulsion increased. UCS of the RC sample increased about 1.7 times whenever the amounts of emulsion were doubled. By contrast, the JS sample showed the highest UCS of 1020 kPa at 2%. The strength decreased over nine times when the emulsion was added as 4% and the shape was not maintained. The sample collapsed at 8% of epoxy emulsion. Based on the results, as the RC sample showed the highest UCS at 8% and the JS sample showed the same at 2%, 4% of emulsion was chosen for the RS samples.

### 3.3. Effect of Acrylic Polymer Aqueous Solution

**Figure 9:**
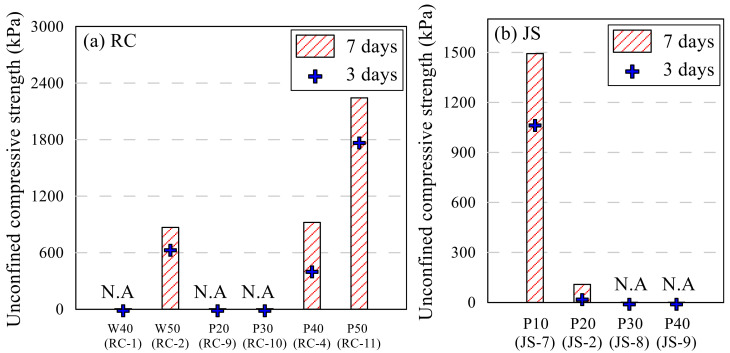
Effect of amount of the polymer aqueous solution on UCS: RC sample (**a**) and JS sample (**b**).

Figure 9 shows the effect of the amount of polymer aqueous solution. Water was also compared with the polymer because of the general characteristics of clay. The strength of the RC appeared at the sample made with 50 g of water, 40 or 50 g of polymer aqueous solution (RS-2/3/11). The UCS of every sample increased over the curing time because of the drying characteristics of clay. Polymer aqueous solution showed higher strength than water. The UCS of the RC made with 50 g of polymer aqueous solution sample was found to be 2243 kPa, which was the highest strength among the RC samples. By contrast, the JS sample showed the highest strength at 10 g of the polymer aqueous solution; the UCS was also the highest among the JS samples. This study evaluated the ratio of epoxy–hardener, amount of epoxy emulsion, and amount of the polymer aqueous solution. It is concluded that the amount of the solution effected to the UCS the most among them. Furthermore, this study used a 5 wt% polymer aqueous solution, which is a small amount. By adding 0.5g of polymer and 9.5g of water, the UCS increased from 921 kPa to 2243 kPa. Thus, it is observed that the UCS of RC and JS both samples were most affected by a small amount of the acrylic polymer, adjusting the amount of the aqueous solution cloud to be the most efficient method to increase and decrease the UCS. For the RS samples, 40 g of aqueous solution was selected.

### 3.4. Red Clay Sample Containing the Sand and Lime

**Figure 10:**
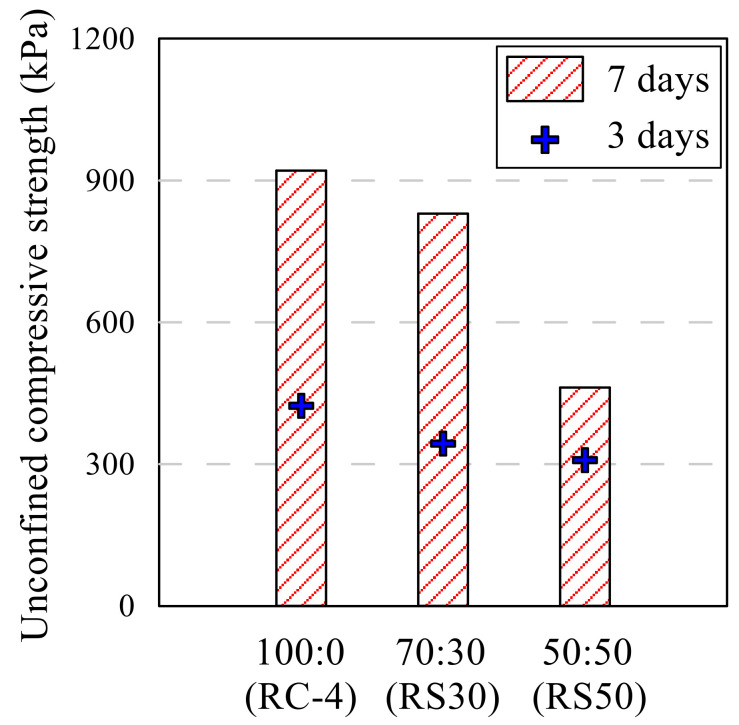
UCS of red clay–sand mixture samples.

**Figure 11:**
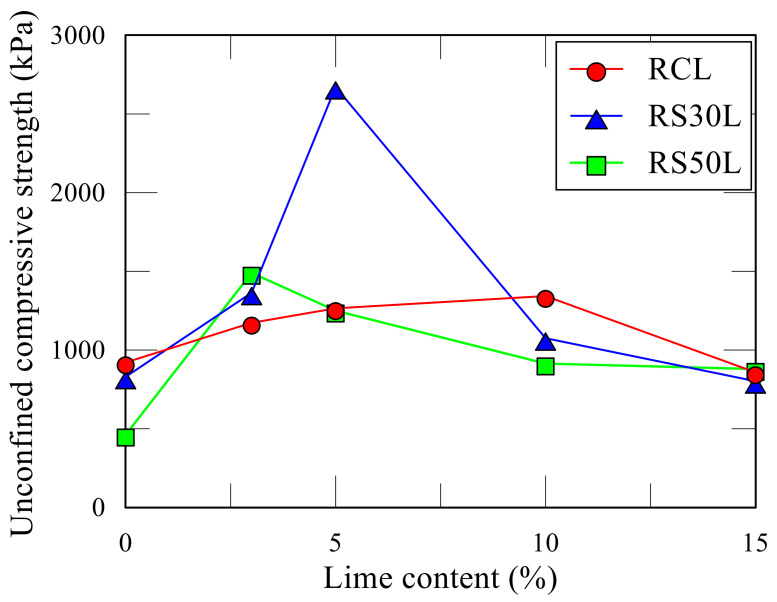
UCS of red clay–sand mixture samples according to the lime.

**Figure 12:**
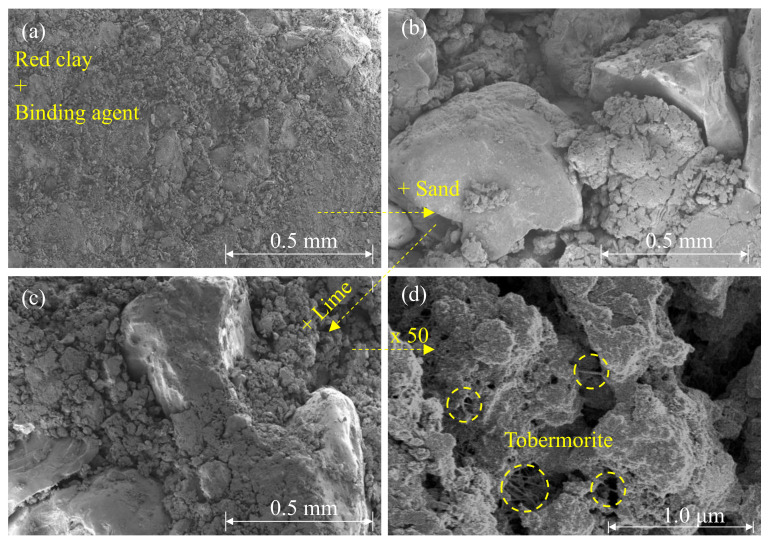
Microstructure images of RC-4 (**a**), RS30 (**b**), and R30L-2 (**c**,**d**).

**Figure 13:**
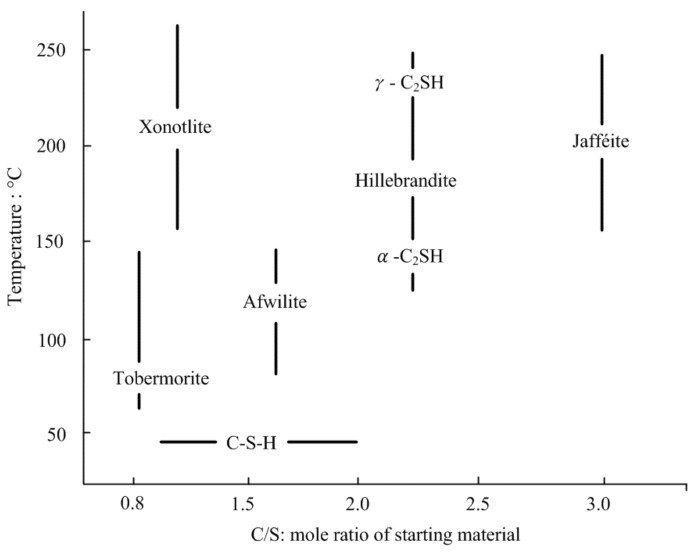
C-S H depending on the calcium oxide/silicon dioxide ratio at various temperatures [37].

**Figure 14:**
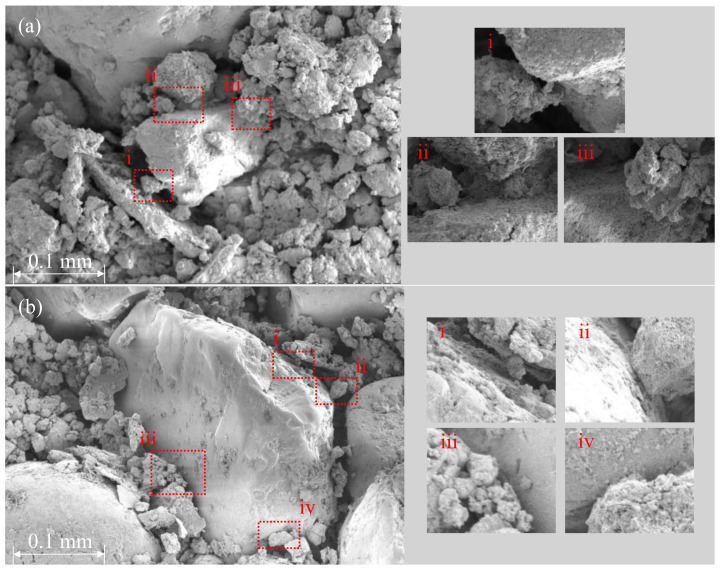
The connection of sand particle and red clay lumps: RC30L-2 sample (**a**) and RC50L-2 sample (**b**).

Table 9 shows the compression test results of the RC and RS samples, and lime added sample. Figure 10 showed the UCS of RC-4, RS30, and RS50. The maximum strength was observed with the RS30L-2 sample made with 70:30 of the soil mixture and 5% of lime. UCS of the RS30L-2 sample for 7 days’ curing was 2671 kPa, and this value was three times that of RC-3, which did not contain the sand and lime. It was observed that sand reduced the strength of the sample without lime, however, when it was added with lime, the strength increased greatly. The UCS according to the lime content was illustrated in Figure 11. Each line in Figure 11 shows a different ratio of red clay and sand and has a peak point at a different lime content. The highest UCS of the RCL sample was observed with 10% of lime content, but those of RS30L and RC50L were at 5% both. Figure 12 shows the SEM images of particles of RC-4 (a), RS30 (b), RS30L-2 (c,d) at the micro scale. It was seen that the lime in the soil reacted with the moisture of the polymer aqueous solution through Figure 12. In the (a) of Figure 12, red clay particles were bonded by the binding agent and arranged by compacting. Furthermore, the bonding area was reduced by adding the sand, because the particles of sand were larger than those of red clay. Therefore, soil particles were bonded efficiently with the same amount of binder, and RC particles which could not bond well lumped and disappeared a lot at (b) compared with that at (a). Additionally, the gap between sand particle and red clay lump became narrow and the connection of the particles was enhanced well at (c). (d) is an image scaled up 100 times of (c), there are many thread-shaped crystals between the particles. These crystals are called tobermorite. A tobermorite crystal is formed by providing lime (Ca^2+^); it is one of calcium silicate hydrate [36], which reacted at a low temperature. There are many formations of calcium silicate hydrate; a formation is affected by the temperature and mole ratio [37]. Figure 13 illustrates various formations of calcium silicate hydrate, according to the temperature and mole ratio. The effect of the clay–sand ratio on the lime added sample can be explained through Figure 14, which shows two SEM images, RS30-2 (a) and RS50-2 (b). Each image shows the microstructure of the highest strength sample of their type. Though the sand particle of the RS50L-2 sample was surrounded more with red clay lumps than that of RC30L-2, a tightly bonded structure was observed more at the RS30L-2 sample image (i-iii). There were some gaps between the sand particle and surrounded lumps (i–iv), and these gaps reduced the UCS. From the results, 30% of sand and 5% of lime were the optimal components to make the best strength of the RC with 40-11-3 of the binding agent.

## 4. Conclusions

This study investigated the strength of the red clay and sand sample made with acrylic polymer aqueous solution and epoxy emulsion. Three types of samples were made and compared: the red clay sample (RC), the sand sample (JS), and the mixture of red clay and sand (RS) sample. Additionally, the lime was added to some of the RC and RS samples, and it was named as RCL and RSL. The highest strength was obtained from the RSL sample, which contained 5% lime. Detailed conclusions are the following.

1. The strength of the RC sample decreased when the epoxy–hardener ratio was higher or lower based on 11:6. The UCS of the RC sample was a 1376 kPa at 11:6 ratio. In the case of the JS sample, the total amount of polymer aqueous solution and epoxy emulsion affected the UCS more than the ratio of epoxy–hardener.
2. The RC sample which contained 8% of the epoxy emulsion showed as 1601 kPa of UCS. In contrast, the JS sample that contained 2% of the epoxy emulsion showed 1020 kPa as the UCS. As the epoxy emulsion was included from 2% to 8% in the sample, the strength of RC sample increased, but the strength of JS sample decreased.
3. The RC sample showed the highest UCS as 2243 kPa with 50 g of polymer aqueous solution, and the JS sample showed the highest UCS as 1493 kPa at 10 g of the solution. Adjusting the amount of the polymer aqueous solution was the most efficient method to get the highest strength.
4. When the red clay mixed with the sand, the strength of sample decreased. However, when the lime was added, the strength of the sample increased more than RC samples. The lime reacted chemically with the silicate of the sand and formed thread-shaped crystals between the soil particles. The 10% of lime and 5% of lime were the best content to the clay sample and clay–sand sample, respectively.
5. The application of acrylic polymer and epoxy as a ground stabilization binder outperforms in strength. Notably, addition of small amount of lime increases the strength dramatically. However, it is necessary to evaluate several dimensions such as impact of the environment, duration of the strength maintenance, etc.

## Figures and Tables

**Table 1:** Chemical components of Jumunjin sand.

	SiO_2_	Al_2_O_3_	K_2_O	NA_2_O	Fe_2_O_3_	CaO	BaO	Cl	L.O.I.
Component (%)	87.70	6.61	4.03	0.76	0.25	0.11	0.09	0.07	0.38

**Table 2:** Chemical components of the red clay.

	SiO_2_	Al_2_O_3_	K_2_O	Fe_2_O_3_	TiO_2_	MgO	CaO	Pd	Ru	ZrO_2_	L.O.I.
Component (%)	58.79	25.87	1.63	3.99	0.74	0.62	0.13	0.07	0.06	0.04	0.38

**Table 3:** Physical properties of the epoxy.

Model	EEW * (g/eq)	Viscosity (cps@25 °C)	Non-Volatile Content (wt%)
KEM-101-50	450–550	1000–10000	47

* EEW is the epoxy equivalent weight.

**Table 4:** Physical properties of the hardener.

Model	TAV * (mgKOH/g)	Viscosity (cps@25 °C)	AHEW ** (g/eq)	Non-Volatile Content (wt%)
KH-700	190–250	3000–10000	170	80

* TAV is the total amine value, ** AHEW is the amine hydrogen equivalent weight.

**Table 5:** Components of the red clay sample.

ID	Polymer Aqueous Solution (g)	Epoxy (g)	Hardener (g)	Water (g)
RC-1	-	-	-	40
RC-2	-	-	-	50
RC-3	40	11	1.5	-
RC-4	40	11	3	-
RC-5	40	11	6	-
RC-6	40	11	9	-
RC-7	40	5.5	1.5	-
RC-8	40	22	6	
RC-9	20	11	3	-
RC-10	30	11	3	
RC-11	50	11	3	-

**Table 6:** Components of the sand sample.

ID	Polymer Aueous Solution (g)	Epoxy (g)	Hardener (g)
JS-1	20	11	1.5
JS-2	20	11	3
JS-3	20	11	6
JS-4	20	11	9
JS-5	20	5.5	1.5
JS-6	20	22	6
JS-7	10	11	3
JS-8	30	11	3
JS-9	40	11	3

**Table 7:** Components of the red clay and sand mixture with lime.

ID	Polymer Solution(g)–Epoxy(g)–Hardener(g)	Red Clay (g)	Sand (g)	Lime (g (%))
RC-4	40-11-3	350	-	-
RS30		245	105	
RS50		175	175	
RCL-1		350	-	10.5 (3)
RCL-2				17.5 (5)
RCL-3				35.0 (10)
RCL-4				52.5 (15)
RS30L-1		245	105	10.5 (3)
RS30L-2				17.5 (5)
RS30L-3				35.0 (10)
RS30L-4				52.5 (15)
RS50L-1		175	175	10.5 (3)
RS50L-2				17.5 (5)
RS50L-3				35.0 (10)
RS50L-4				52.5 (15)

**Table 8:** UCS results of the RC and JS samples.

ID	Polymer Aqueous Solution–Epoxy–Hardener	3 Days		7 Days	
		UCS * (kPa)	ε_ucs_ * (%)	UCS * (kPa)	ε_ucs_ * (%)
RC-1	Water 40	N.A.			
RC-2	Water 50	647	1.18	868	1.01
RC-3	40–11–1.5	179	1.29	472	1.07
RC-4	40–11–3	418	1.51	921	1.58
RC-5	40–11–6	843	1.81	1376	1.48
RC-6	40–11–9	654	1.77	820	1.67
RC-7	40–5.5–1.5	387	0.95	540	0.84
RC-8	40–22–6	1027	1.92	1601	1.86
RC-9	20–11–3	N.A.			
RC-10	30–11–3	N.A.			
RC-11	50–11–3	1786	1.89	2243	1.82
JS-1	20–11–1.5	40	2.21	81	2.11
JS-2	20–11–3	29	2.54	108	1.97
JS-3	20–11–6	30	2.48	96	2.04
JS-4	20–11–9	54	2.17	132	2.15
JS-5	20–5.5–1.5	829	1.87	1020	1.74
JS-6	20–22–6	N.A.			
JS-7	10–11–3	1074	1.94	1493	1.81
JS-8	30–11–3	N.A.			
JS-9	40–11–3	N.A.			

* The deviation range of UCS and **ε_ucs_** were ±0.5 on every test.

**Table 9:** UCS results of red clay and sand mixture sample.

ID	Soil Ratio (Red Clay:Sand)	Lime (%)	3 Days		7 Days	
			UCS * (kPa)	ε_ucs_ * (%)	UCS * (kPa)	ε_ucs_ * (%)
RC-4	100:0	-	432	1.63	921	1.58
RS30	70:30		352	1.85	830	1.65
RS50	50:50		317	1.75	462	1.74
RCL-1	100:0	3	584	0.75	1173	1.87
RCL-2		5	795	1.51	1265	1.55
RCL-3		10	858	1.23	1343	1.77
RCL-4		15	591	1.21	858	1.43
RS30L-1	70:30	3	667	1.71	1364	1.60
RS30L-2		5	1433	1.41	2671	2.03
RS30L-3		10	633	1.47	1075	1.47
RS30L-4		15	401	1.28	802	1.13
RS50L-1	50:50	3	893	1.29	1490	1.73
RS50L-2		5	788	2.32	1250	1.92
RS50L-3		10	717	2.54	914	1.88
RS50L-4		15	717	3.07	879	2.73

* The deviation rrange of UCS and **ε_ucs_** were ±0.5 on every test.

## Data Availability

Data sharing not applicable to this article as no datasets were generated or analysed during the current study.
